# Relationships between pathological factors and long-term outcomes in patients enrolled in two prospective randomized controlled trials comparing the efficacy of oral tegafur–uracil with CMF (N·SAS-BC 01 trial and CUBC trial)

**DOI:** 10.1007/s10549-020-06018-1

**Published:** 2020-12-01

**Authors:** Shinji Ohno, Shigehira Saji, Norikazu Masuda, Hitoshi Tsuda, Futoshi Akiyama, Masafumi Kurosumi, Akihiko Shimomura, Nobuaki Sato, Shintaro Takao, Shozo Ohsumi, Yutaka Tokuda, Hideo Inaji, Toru Watanabe, Yasuo Ohashi

**Affiliations:** 1 Cancer Institute Hospital of Japanese Foundation for Cancer Research, Tokyo, Japan; 2 Fukushima Medical University, Fukushima, Japan; 3 National Hospital Organization Osaka National Hospital, Osaka, Japan; 4 National Defense Medical College, Saitama, Japan; 5 Cancer Institute of the Japanese Foundation for Cancer Research, Tokyo, Japan; 6 Kameda Medical Center, Chiba, Japan; 7 National Cancer Center Hospital, Tokyo, Japan; 8 Niigata Cancer Center Hospital, Niigata, Japan; 9 Hyogo Cancer Center, Hyogo, Japan; 10 NHO Shikoku Cancer Center, Ehime, Japan; 11 Tokai University School of Medicine, Kanagawa, Japan; 12 Kaizuka City Hospital, Osaka, Japan; 13 Hamamatsu Oncology Center, Shizuoka, Japan; 14 Chuo University, Tokyo, Japan

**Keywords:** Tegafur–uracil, CMF, Adjuvant chemotherapy, Breast cancer, Tumor-infiltrating lymphocyte

## Abstract

**Purpose:**

To evaluate the efficacies of cyclophosphamide, methotrexate, and fluorouracil (CMF) and tegafur–uracil (UFT) as adjuvant therapy in patients with resected stage I–IIIA breast cancer by immunohistochemistry (IHC)-based subtype and to determine the relationships between clinicopathological factors and long-term outcomes.

**Methods:**

A pooled analysis of the randomized controlled N·SAS-BC 01 and CUBC studies was conducted. Expression of hormone receptors (HRs; estrogen and progesterone receptors), human epidermal growth factor receptor 2 (HER2), and Ki67were assessed by IHC. Tumor-infiltrating lymphocytes (TILs) and nuclear/histological grades were determined by hematoxylin and eosin staining. Relapse-free survival (RFS) and overall survival (OS) were estimated by Kaplan–Meier analysis and hazard ratios were determined by Cox model adjusted for baseline tumor size and nodal status.

**Results:**

A total of 689 patients (342 CMF and 347 UFT) were included in the analyses with a median follow-up of 11.1 years. There was no significant difference in RFS or OS between the two cohorts (RFS: 0.96 [95% confidence interval: 0.71–1.30], log-rank test *p* = 0.80; OS: 0.93 [0.64–1.35], *p* = 0.70). There was no difference in RFS or OS between the two cohorts for HR+/HER2− and HR+/HER2+ subtypes. RFS was significantly longer in patients treated with UFT compared with CMF in patients with HR−/HER2+ subtype (0.30 [0.10–0.88], *p* = 0.03). A high TILs level was associated with a better OS compared with low TILs level (*p* = 0.02).

**Conclusions:**

This long-term follow-up study showed that RFS and OS were similar in patients with luminal-type breast cancer treated with CMF and UFT.

**Electronic supplementary material:**

The online version of this article (10.1007/s10549-020-06018-1) contains supplementary material, which is available to authorized users.

## Introduction

The Japanese Collaborative Study Group of Adjuvant Chemoendocrine Therapy for Breast Cancer (ACETBC) established adjuvant endocrine therapy with the oral fluoropyrimidine tegafur–uracil (UFT) to treat patients with early-stage breast cancer based on meta-analyses including studies conducted between 1988 and 1995 [1, 2]. However, the standard postoperative adjuvant chemotherapy in the European Union and the USA at that time was cyclophosphamide, methotrexate, and fluorouracil (CMF), and the National Surgical Adjuvant Study for Breast Cancer (N·SAS-BC) 01 and Comparative Trial of UFT + tamoxifen (TAM) with CMF + TAM in Adjuvant Therapy for Breast Cancer (CUBC) studies were, therefore, initiated in 1996 to demonstrate the non-inferiority of UFT compared with CMF in patients with resected stage I–IIIA breast cancer [3, 4]. Although neither study demonstrated non-inferiority because the target number of patients was not reached, a pooled analysis combining these studies that was conducted to overcome this issue showed that UFT was non-inferior to CMF in estrogen receptor-positive (ER+) patients [5]. Furthermore, a recent study in which the patients in these two studies were followed up for over 10 years demonstrated that the Kaplan–Meier curves for these two regimens for relapse-free survival (RFS) and overall survival (OS) appeared to be superimposable [6].

A treatment choice for breast cancer has recently been determined based on the current standard clinicopathological biomarkers, including the expression levels of hormone receptors (HRs; ER and progesterone receptor [PgR]), human epidermal growth factor receptor 2 (HER2), and Ki67 [7]. However, these markers were not completely established when the N·SAS-BC 01 and CUBC studies were initiated in 1996, and the findings are thus not fully applied in clinical practice. We therefore examined the expression of ER, PgR, HER2, Ki67, and tumor-infiltrating lymphocytes (TILs), as well as nuclear and histological grades, in tumor specimens from patients enrolled in these two studies using the currently available measurement methods.

In this cross-sectional, observational, pooled study, we evaluated the efficacies of adjuvant CMF and UFT according to immunohistochemistry (IHC)-based intrinsic subtypes and the relationships between the clinicopathological factors and updated long-term follow-up prognostic outcomes in patients with resected stage I–IIIA breast cancer enrolled in the randomized N·SAS-BC 01 and CUBC studies to identify a subset of patients responsive to UFT and CMF.

## Materials and methods

This study (UMIN000022571) was approved by the independent ethics committee at each study site and conducted in agreement with the Helsinki Declaration and ethical guidelines for medical research on humans. Written consent was obtained from patients before initiating any process in this study. If written consent could not be obtained because a patient had died or could not be reached, samples were sent to a central pathology review office after confirmation by each independent ethics committee that the various provisions based on the Ethical Guidelines for Medical and Health Research Involving Human Subjects had been met.

### Previous N·SAS-BC 01 and CUBC studies

Details of the N·SAS-BC 01 and CUBC studies and subsequent analyses have been described previously [3–5]. In brief, patients were enrolled in the N·SAS-BC 01 and CUBC studies from October 1996 to April 2001 and from September 1996 to July 2000, respectively. Both studies included patients with resected stage I–IIIA breast cancer, irrespective of their HR status, but the N·SAS-BC 01 study enrolled high-risk (invasive ductal carcinoma with invasive size ≥ 5 mm and grade 2 or 3, or invasive lobular carcinoma, or metaplastic carcinoma), node-negative, and the CUBC study enrolled node-positive patients (ranged 1–9). Patients in both studies were treated with 6 cycles of either CMF or UFT for 2 years. Patients in the CUBC study also received TAM for 2 years, irrespective of their HR expression status, and patients with ER+, PgR+, or both in the N·SAS-BC 01 study were treated with TAM for 5 years. No patient was treated with trastuzumab in the two studies, and radiation therapy was allowed only in the N·SAS-BC 01 study. Protein expression levels of ER and PgR to assess patient characteristics were determined by enzyme immunoassay at each participating hospital.

### Patients

Patients included in the previous N·SAS-BC 01 and CUBC studies with available paraffin-embedded specimens of surgically excised tumor tissue and for whom use of the specimens had been authorized by the applicable independent ethics committee were included in this study. Patients who declined consent for use of their samples were excluded from the study.

### Detection and evaluation of clinicopathological factors and prognostic outcomes

The study outcomes were RFS and OS according to HR/HER2 subtypes, and the relationships between clinicopathological factors, including age, tumor size, HR, HER2, Ki67, histological grade and TILs, and updated long-term follow-up prognostic outcomes (RFS and OS) in patients treated with CMF and UFT. In this study, RFS was defined as the period from the date of randomization to the last-confirmed date of no recurrence or of death from any cause, and OS was defined as the period from the date of randomization to the date of death from any cause with a cut-off date of June 30, 2018.

Protein expression levels of ER, PgR, HER2, Ki67 and TILs, as well as nuclear and histological grades, were assessed in the paraffin-embedded sections from each patient and evaluated by central pathological review. ER, PgR, and Ki67 protein expression were detected by immunohistochemistry with monoclonal antibodies SP1, 1E2 (Roche Diagnostics K.K., Japan), and MIB-1 (DAKO Japan Agilent, Japan), respectively. ER and PgR protein expression levels were determined as the sum of the proportion score (0–5) and intensity score (0–3), and ER+ and PgR+ were defined as total scores of 3–8 and ER− and PgR− as total scores of 0–2. Based on the expression results, HR positivity (HR +) was defined as ER+ and/or PgR+, and HR negativity (HR−) as ER− and PgR−.

HER2 expression was detected by semi-quantitative IHC assay (DAKO HercepTest II, Agilent, Japan; score 0–3+) in samples from all patients, with further fluorescence in situ hybridization (FISH) analysis in those with 2+ staining (PathVysion HER-2 DNA Probe Kit; Abbott, Japan). HER2 negativity (HER2−) was defined as an IHC score of 0–2 and negative FISH, and HER2-positive (HER2+) as an IHC score of 2 or 3 and positive FISH results. Nuclear grade (grades 1–3) [8, 9], and histological grade (grades 1–3) [10] were evaluated in hematoxylin and eosin-stained sections. Expression of TILs was evaluated according to the criteria of International TILs Working Group 2014 [11–13]. Briefly, using an optical microscope under × 200 and × 400 magnifications, the panel pathologists classified TILs levels into the following three grades: low < 10%, intermediate ≥ 10 to ≤ 40%, and high > 40%.

### Statistical methods

Summary statistics, number of patients, mean, standard deviation, minimum, median, and maximum values were obtained for the patients’ baseline characteristics. Between-cohort differences were evaluated using *χ*^2^ test and *t* test. RFS and OS were estimated using Kaplan–Meier analysis, and differences between the two treatment cohorts were tested by the log-rank test. Evaluations of RFS and OS in subgroups were adjusted for clinical characteristics chosen based on the selection criterion set at *α* = 0.15, namely tumor size (< 3 and ≥ 3 cm) and nodal status (0 and ≥ 1). Hazard ratios and 95% confidence intervals (CIs) were determined using univariate unadjusted Cox proportional hazards model to evaluate the prognostic factors and were summarized in forest plots. Statistical significance was set at a two-sided *p* < 0.05. Statistical analysis was performed using SAS version 9.4 or higher (SAS Institute, Inc., Cary, NC, USA).

## Results

### Patients

**Fig. 1:**
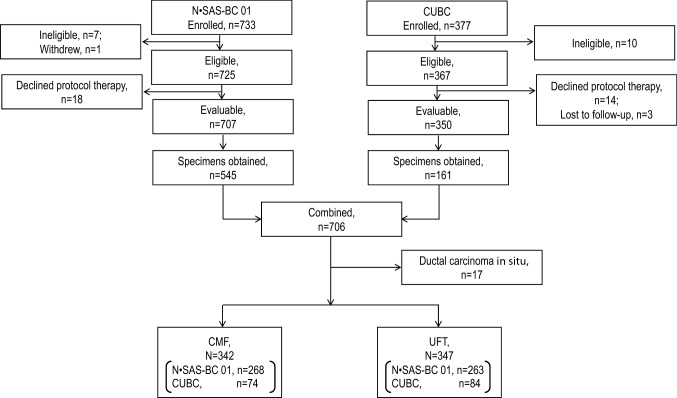
Patient flow diagram in the previous N·SAS-BC 01 [3] and CUBC study [4] and the present study. *CMF* cyclophosphamide, methotrexate, and fluorouracil, *CUBC* comparative trial of UFT + tamoxifen with CMF + tamoxifen in adjuvant therapy for breast cancer, *N·SAS-BC 01* National Surgical Adjuvant Study for Breast Cancer, *UFT* tegafur-uracil

The study was conducted from December 2015 to November 2018. A total of 1057 patients enrolled and analyzed in the N·SAS-BC 01 and CUBC studies were involved in this pooled analysis (Fig. 1), including 707 patients in the N·SAS-BC 01 and 350 patients in the CUBC studies. Paraffin-embedded pathology specimens were available for 545 and 161 of the study patients, respectively. Seventeen patients were excluded from the study because submitted pathology specimens were ductal carcinoma in situ component, and 689 patients (342 treated with CMF and 347 treated with UFT) were therefore included in the final analyses. Characteristics of evaluable patients in the N·SAS-BC 01 and CUBC studies and those included in the present study are summarized in Supplementary Table 1.

The patients treated with CMF and UFT were well balanced in terms of age, tumor size, nodal stage, tumor subtype, Ki67, and TILs expression, and nuclear and histological grades (Table 1). Patients divided according to subtypes were also well balanced (Supplementary Table 2).

**Table 1:** Patient characteristics

Variable	CMF (N = 342)	UFT (N = 347)	*p* value
Patients, n (%)			
CUBC	74 (21.6)	84 (24.2)	0.422^a^
N·SAS-BC 01	268 (78.4)	263 (75.8)	
Age (years)			0.995^b^
Median (range)	53.0 (32–74)	53.0 (32–75)	
< 50, n (%)	133 (38.9)	134 (38.6)	0.942^a^
≥ 50	209 (61.1)	213 (61.4)	
Tumor size (cm)			0.454^b^
Median (range)	2.1 (0.5–8.0)	2.2 (0.5–10.0)	
< 3 cm, n (%)	257 (75.1)	247 (71.2)	0.240^a^
≥ 3 cm	85 (24.9)	100 (28.8)	
Lymph nodes, n (%)			0.717^a^
0	268 (78.4)	263 (75.8)	
1–3	61 (17.8)	70 (20.2)	
4	13 (3.8)	14 (4.0)	
Histological type, n (%)			0.204^a^
Invasive ductal	316 (92.4)	311 (89.6)	
Other	26 (7.6)	36 (10.4)	
Estrogen receptor, n (%)			0.309^a^
Positive	247 (72.2)	237 (68.3)	
Negative	94 (27.5)	107 (30.8)	
Unknown	1 (0.3)	3 (0.9)	
Progesterone receptor, n (%)			0.890^a^
Positive	203 (59.4)	203 (58.5)	
Negative	138 (40.4)	141 (40.6)	
Unknown	1 (0.3)	3 (0.9)	
Human epidermal growth factor receptor 2, n (%)			0.106^a^
Positive	72 (21.1)	55 (15.9)	
Negative	269 (78.7)	283 (81.6)	
Unknown	1 (0.3)	9 (2.6)	
Subtype, n (%)			0.155^a^
HR+/HER2−	210 (61.4)	210 (60.5)	
HR−/HER2−	59 (17.3)	73 (21.0)	
HR+/HER2+	42 (12.3)	26 (7.5)	
HR−/HER2+	29 (8.5)	29 (8.4)	
Unknown	2 (0.6)	9 (2.6)	
Ki67 (%)			0.619^b^
Median (range)	22.6 (0.0–97.2)	22.3 (0.0–86.0)	
< 20%	146 (42.7)	160 (46.1)	0.366^a^
≥ 20%	196 (57.3)	187 (53.9)	
Nuclear grade, n (%)			0.624^a^
Grade 1	89 (26.0)	90 (25.9)	
Grade 2	80 (23.4)	91 (26.2)	
Grade 3	172 (50.3)	163 (47.0)	
Unknown	1 (0.3)	3 (0.9)	
Histological grade, n (%)			0.495^a^
Grade 1	38 (11.1)	30 (8.6)	
Grade 2	146 (42.7)	158 (45.5)	
Grade 3	157 (45.9)	156 (45.0)	
Unknown	1 (0.3)	3 (0.9)	
Tumor-infiltrating lymphocytes, n (%)			0.359^a^
Low	228 (66.7)	246 (70.9)	
Intermediate	74 (21.6)	66 (19.0)	
High	39 (11.4)	31 (8.9)	
Unknown	1 (0.3)	4 (1.2)	

^a^χ^2^ test

^b^*t* test

*CMF* cyclophosphamide, methotrexate, and fluorouracil, *UFT* tegafur-uracil, *HER2* human epidermal growth factor receptor 2, *HR* hormone receptor

### RFS and OS in all patients and according to HR/HER2 subtypes

**Fig. 2:**
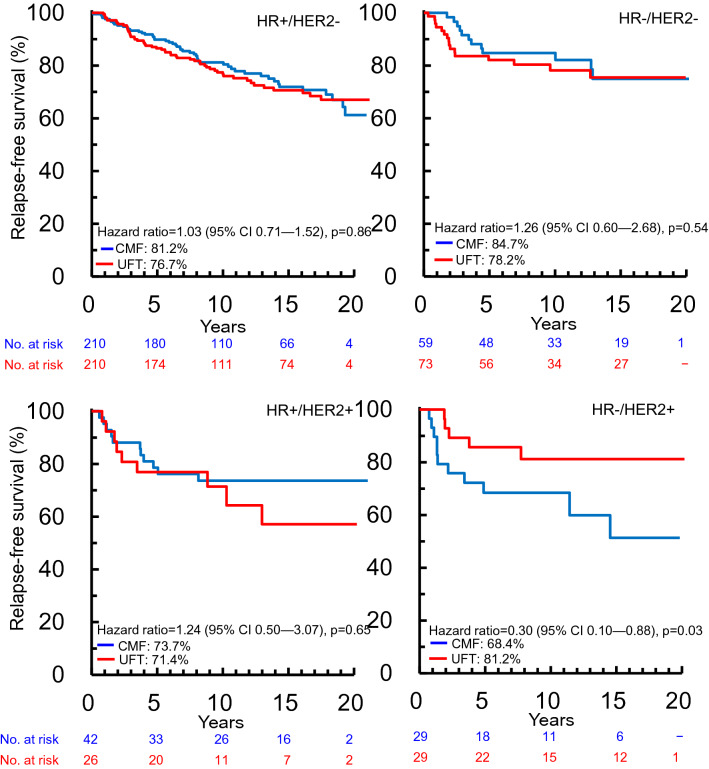
Kaplan–Meier survival curves for relapse-free survival adjusted for tumor size and nodal status in patients stratified according to HR/HER2 subtype. *CI* confidence interval, *CMF* cyclophosphamide, methotrexate, and fluorouracil, *HER2* human epidermal growth factor receptor 2, *HR* hormone receptor, *UFT* tegafur–uracil

The median follow-up time as of December 31, 2015 was 11.1 years (12.1 years in the N·SAS-BC 01 trial and 8.3 years in the CUBC trial) [6]. The Kaplan–Meier RFS curves for the CMF and UFT cohorts in all patients and those included in the final analysis are presented in Supplementary Fig. 1. There was no significant difference in RFS between the two cohorts for patients included in the final analysis (*p* = 0.80). RFS analysis by HR and HER2 receptor subtypes showed that patients with HR−/HER2+ subtype treated with UFT had a significantly higher RFS than those treated with CMF (*p* = 0.03). However, there was no significant difference in RFS between the treatment cohorts for any other HR/HER2 subtypes (*p* > 0.50, Fig. 2).

**Fig. 3:**
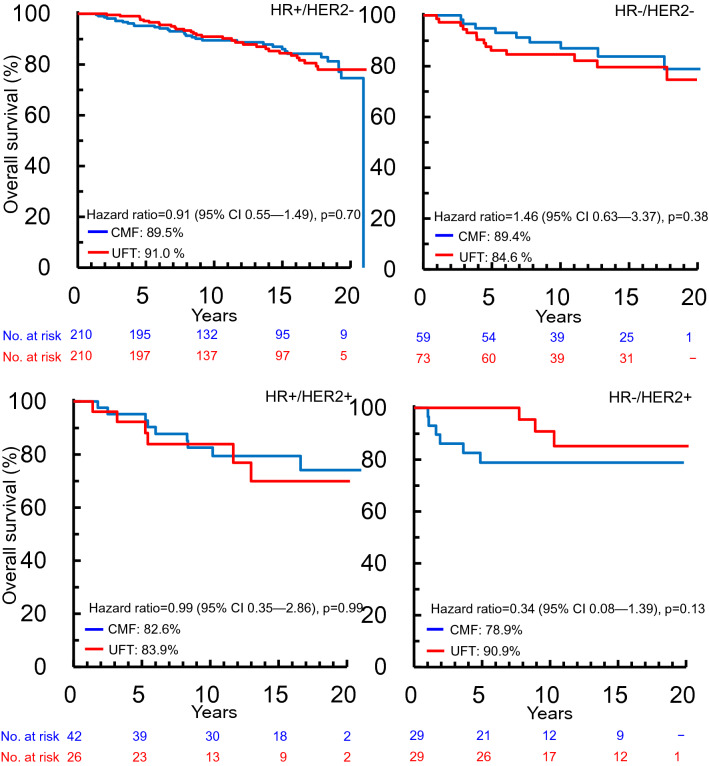
Kaplan–Meier survival curves for overall survival adjusted for tumor size and nodal status in patients stratified according to HR/HER2 subtype. *CI* confidence interval, *CMF* cyclophosphamide, methotrexate, and fluorouracil, *HER2* human epidermal growth factor receptor 2, *HR* hormone receptor, *UFT* tegafur–uracil

There was no significant difference in OS between the two cohorts for patients included in the final analysis (*p* = 0.70, Supplementary Fig. 1). OS analysis by HR and HER2 receptor subtypes showed that patients with HR−/HER2+ subtype treated with UFT had a numerically higher OS than those treated with CMF. However, there was no significant difference in OS between the treatment cohorts for any HR/HER2 subtype (*p* > 0.13, Fig. 3).

### Subgroup analyses of RFS in patients included in the final analysis and according to HR/HER2 subtypes

**Fig. 4:**
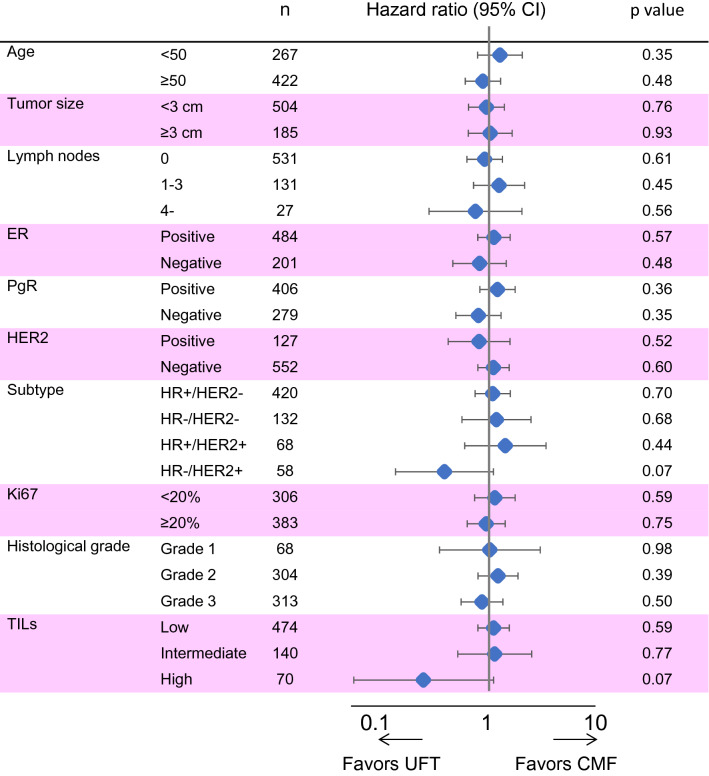
Forrest plot for subgroup analyses of relapse-free survival in patients included in the final analysis. *CMF* cyclophosphamide, methotrexate, and fluorouracil, *ER* estrogen receptor, *PgR* progesterone receptor, *TILs* tumor-infiltrating lymphocyte, *UFT* tegafur–uracil

Subgroup analyses according to baseline characteristics showed no significant differences in the RFS between the two treatment cohorts (Fig. 4). Although it did not reach statistical significance, RFS in patients treated with UFT tended to be longer in patients with HR−/HER2+ subtype (hazard ratio 0.38 [0.13–1.09], *p* = 0.07) and in patients with high TILs levels (hazard ratio 0.24 [0.05–1.10], *p* = 0.07).

**Fig. 5:**
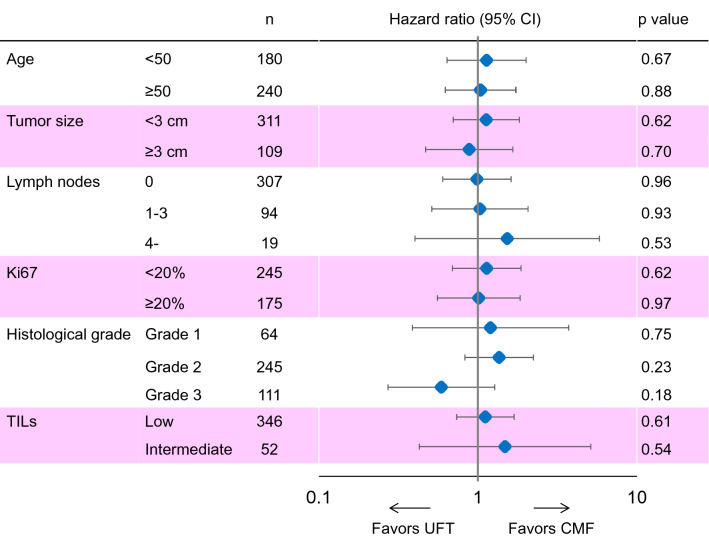
Forrest plot for subgroup analyses of relapse-free survival in patients with HR+/HER2− subtype. *CMF* cyclophosphamide, methotrexate, and fluorouracil, *TILs* tumor-infiltrating lymphocyte, *UFT* tegafur–uracil

Similarly, baseline characteristics had no significant impact on the RFS following CMF or UFT treatment in patients with HR+/HER2− subtype (Fig. 5).

Subgroup analyses showed no notable trend in OS between the two treatment cohorts in patients included in the final analysis or in those stratified according to HR/HER2 subtypes (data not shown).

### Analysis of TILs expression levels as a prognostic factor

**Fig. 6:**
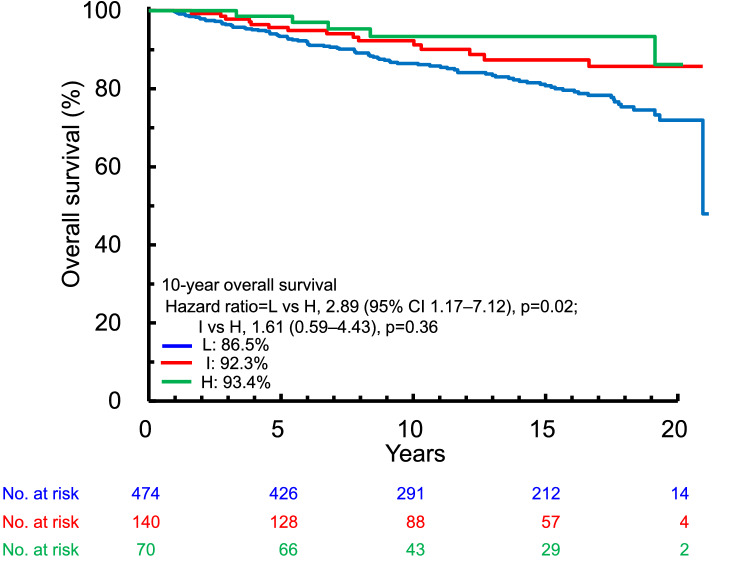
Overall survival according to tumor-infiltrating lymphocyte level (low, intermediate, high) in patients included in the final analysis. *CI* confidence interval, *H* high, *I* intermediate, *L* low

At 10 years, there were 59, 10, and 4 OS events, and the OS rates were 86.5% (95% CI 82.9–89.4), 92.3% (86.1–95.8), and 93.4% (83.2–97.5) in patients with low, intermediate, and high TILs levels, respectively. Among patients included in the final analysis, patients with high TILs levels showed a significantly improved OS compared to those with low TILs levels (*p* = 0.02, Fig. 6).

**Fig. 7:**
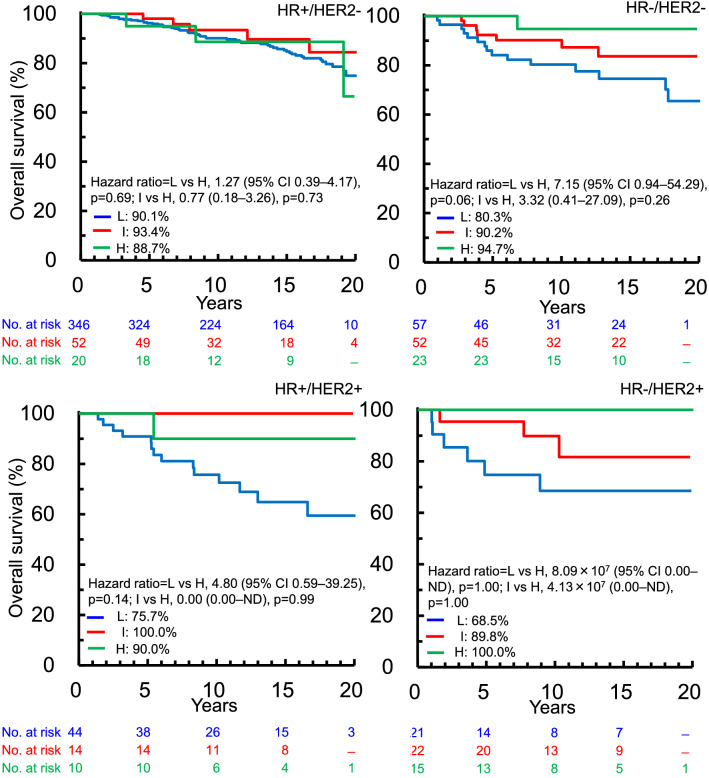
Kaplan–Meier survival curves for overall survival according to tumor-infiltrating lymphocyte level (low, intermediate, high) adjusted for tumor size and nodal status in patients stratified according to HR/HER2 subtype. *CI* confidence interval, *H* high, *HER2* human epidermal growth factor receptor 2, *HR* hormone receptor, *I* intermediate, *L* low, *ND* not determined

Similar trends in OS were found in patients with HR−/HER2− and HR−/HER2+ breast cancer, but no difference was detected in HR+/HER2− subtype irrespective of TILs levels (Fig. 7).

## Discussion

The present study re-evaluated the efficacies of UFT and CMF using a pooled analysis of randomized controlled studies and newly obtained clinicopathological data and updated long-term prognosis outcomes. Although the statistical power was not sufficient to test the non-inferiority, our results showed similar efficacies of UFT and CMF in patients with luminal-type breast cancer. This study was also clinically significant because it demonstrated the efficacies of UFT and CMF in preventing late recurrence in Japanese patients with luminal-type breast cancer, which is a major clinical concern [14].

The efficacy of UFT determined by RFS and OS was similar to that of CMF in patients with HR+/HER2− and HR+/HER2+ subtypes and was significantly better in patients with HR−/HER2+ subtype. These results indicate that UFT is effective in patients with these subtypes, and its less toxic nature suggests that it may be a suitable option for patients who are intolerant of standard therapy. To the best of our knowledge, this is the first study demonstrating a better prognosis with UFT in patients with HR−/HER2+ subtype. However, further studies with larger sample sizes are needed to verify our results and to clarify the mechanism responsible for the better prognosis in patients with this specific subtype. Although the differences were not significant, both RFS and OS were slightly shorter in the UFT compared with the CMF cohort patients with HR−/HER2− subtype. Further studies are therefore needed to find more suitable therapeutic options for patients with this subtype.

Our results showed that high levels of TILs were associated with a favorable prognosis among the patients included in the final analysis and among patients with HR−/HER2− and HR−/HER2+ subtypes that are consistent with previous studies [15–17]. However, the results showed no effect of TILs level on prognosis in patients with HR+/HER2− subtype, suggesting that TILs may play a different role in these patients compared with patients with HR+ subtypes. Regarding the HR+/HER2+ subtype, more samples are needed to draw a solid conclusion about this relationship. The interactions between tumor cells and immune cells have only recently become a focus of investigations, and information on the relationship between TILs and prognostic outcomes is still lacking. However, given that chemotherapy and adjuvant therapy also affect the immune system, the current data including over 10 years of follow-up data and clinicopathological factors correlated at an individual patient level are expected to improve our understanding of such interactions.

This study had some limitations. First, all patients in the CUBC study were treated with TAM, irrespective of their HR expression status, for 2 years. However, the current recommendation is that only HR+ patients should receive TAM for > 5 years. The potential impact of 2-year rather than 5-year TAM treatment on RFS and OS in HR+ patients in the CUBC study thus needs to be taken into consideration when interpreting the present results. Second, treatment regimens investigated in the previous N·SAS-BC 01 and CUBC studies differ from the current standard therapy. For example, no patients with HER2+ in the N·SAS-BC 01 and CUBC studies were treated with trastuzumab, because trastuzumab had not yet been approved when these studies were carried out. In addition, the current standard postoperative adjuvant chemotherapy consists of anthracyclines or taxanes, whereas CMF therapy is currently determined as useful in certain circumstances only. Furthermore, the previous Trial Assigning Individualized Options for Treatment (TAILORx) demonstrated no benefit of adding chemotherapy to hormone therapy in the majority of node-negative HR+/HER2− breast cancer patients, determined based on the 21-gene recurrence score 11–25 using Oncotype DX [18], indicating that the present study included patients who may not benefit from adjuvant chemotherapy. A future study is needed to compare the efficacy of UFT with the current standard therapy using real-world data, to identify a patient population that responds favorably to the UFT therapy. Third, tumor sections were collected from 708 out of 1057 patients, which suggests a potential bias in the sample collection. Indeed, a significant difference in the number of lymph nodes was found between eligible patients in the previous two studies and those included in this study (Supplementary Table 1, *p* < 0.0001) because more specimens were collected in patients in the N·SAS-BC 01 study than the CUBC study. However, no significant difference was found between two treatment cohorts in the present study.

Breast cancer subtypes have been studied based on a set of gene expression patterns, and commercially available genomic tests, such as Oncotype DX, have been effectively utilized to determine the tailored treatment for each patient’s cancer subtype [18, 19]. Further studies using genomic tests are expected to identify a set of recurrence risk factors and to find which risk factors respond to UFT therapy.

In conclusion, the present pooled analysis of two randomized controlled studies demonstrated that RFS and OS in the UFT and CMF cohorts were similar in patients with luminal-type breast cancer.

## Electronic supplementary material

Below is the link to the electronic supplementary material.

Electronic supplementary material 1 (PDF 254 kb)

## Data Availability

Data generated or analyzed during this study are on file with and are not publicly available.

## Abbreviations

CI: Confidence interval
CMF: Cyclophosphamide, methotrexate, and 5-fluorouracil
CUBC: Comparative Trial of UFT + TAM with CMF + TAM in Adjuvant Therapy for Breast Cancer
ER: Estrogen receptor
FISH: Fluorescence in situ hybridization
HER2: Human epidermal growth factor receptor 2
HR: Hormone receptor
N·SAS-BC: National Surgical Adjuvant Study for Breast Cancer
OS: Overall survival
PgR: Progesterone receptor
RFS: Relapse-free survival
TAM: Tamoxifen
TILs: Tumor-infiltrating lymphocytes
UFT: Tegafur–uracil
