# Supplementary material for: Relationships between pathological factors and long-term outcomes in patients enrolled in two prospective randomized controlled trials comparing the efficacy of oral tegafur–uracil with CMF (N·SAS-BC 01 trial and CUBC trial)
Source: Breast Cancer Res Treat. 2020 Dec 1;186(1):135–47. doi: 10.1007/s10549-020-06018-1 (PMC7940338; doi:10.1007/s10549-020-06018-1)
Supplement: Supplementary file 1 — Electronic supplementary material 1 (PDF 254 kb) [file 10549_2020_6018_MOESM1_ESM.pdf]

Supplementary material

Relationships between pathological factors and long-term outcomes in patients enrolled in two prospective randomized controlled trials comparing the efficacy of oral tegafur-uracil with CMF (N•SAS-BC 01 trial and CUBC trial)

Journal name: Breast Cancer Research and Treatment

Shinji Ohno[1] • Shigehira Saji[2] • Norikazu Masuda[3] • Hitoshi Tsuda[4] • Futoshi Akiyama[5] • Masafumi Kurosumi[6] • Akihiko Shimomura[7] • Nobuaki Sato[8] • Shintaro Takao[9] • Shozo Ohsumi[10] • Yutaka Tokuda[11] • Hideo Inaji[12] • Toru Watanabe[13] • Yasuo Ohashi[14]

[1] Cancer Institute Hospital of Japanese Foundation for Cancer Research, Tokyo, Japan

[2] Fukushima Medical University, Fukushima, Japan

[3] National Hospital Organization Osaka National Hospital, Osaka, Japan

[4] National Defense Medical College, Saitama, Japan

[5] Cancer Institute of the Japanese Foundation for Cancer Research, Tokyo, Japan

[6] Kameda Medical Center, Chiba, Japan

[7] National Cancer Center Hospital, Tokyo, Japan

[8] Niigata Cancer Center Hospital, Niigata, Japan

[9] Hyogo Cancer Center, Hyogo, Japan

[10] NHO Shikoku Cancer Center, Ehime, Japan

[11] Tokai University School of Medicine, Kanagawa, Japan

[12] Kaizuka City Hospital, Osaka, Japan

[13] Hamamatsu Oncology Center, Shizuoka, Japan

[14] Chuo University, Tokyo, Japan

Corresponding author: Shinji Ohno

e-mail: shinji.ohno@jfcr.or.jp

Supplementary Table 1. Characteristics of evaluable patients in the N•SAS-BC 01 and CUBC studies, and in the present study

**Variables**	**N•SAS-BC 01 and CUBC studies[a] (N=1057)**	**The present study (N=689)**	**p value**
CUBC, n (%)	350 (33.1)	158 (22.9)	<0.0001[b]
N•SAS-BC 01, n (%)	707 (66.9)	531 (77.1)	
Age (years)			0.687[c]
Median (range)	53.0 (31–75)	53.0 (32–75)	
<50, n (%)	416 (39.4)	267 (38.8)	0.800[b]
≥50	641 (60.6)	422 (61.2)	
Tumor size (cm)			0.038[c]
Median (range)	2.2 (0.4–12.0)	2.1 (0.5–10.0)	
<3 cm, n (%)	740 (70.0)	540 (73.1)	0.157[b]
≥3 cm	317 (30.0)	185 (26.9)	
Lymph nodes, n (%)			<0.0001[b]
0	707 (66.9)	531 (77.1)	
1–3	273 (25.8)	131 (19.0)	
4	77 (7.3)	27 (3.9)	
Histological type, n (%)			0.357[b]
Invasive ductal	975 (92.2)	627 (91.0)	
Other	82 (7.8)	62 (9.0)	
Estrogen receptor[d], n (%)			0.200[b]
Positive	534 (50.5)	380 (55.2)	
Negative	479 (45.3)	300 (43.5)	
Unknown	44 (4.2)	9 (1.3)	
Progesterone receptor[d], n (%)			0.358[b]
Positive	485 (45.9)	346 (50.2)	
Negative	501 (47.4)	326 (47.3)	
Unknown	71 (6.7)	17 (2.5)	
Human epidermal growth factor receptor 2, n (%)			

[a]Evaluable patients in the N•SAS-BC 01 (707 patients) and CUBC (350 patients) studies.

[b]χ^2 test

[c]*t*-test

[d]Based on the expression measured locally in the previous N•SAS-BC 01 and CUBC studies.

Supplementary Table 2. Patient characteristics according to HR/HER2 subtype

Variable	HR+/HER2−		HR−/HER2−		HR+/HER2+		HR−/HER2+	
	CMF (N=210)	UFT (N=210)	CMF (N=59)	UFT (N=73)	CMF (N=42)	UFT (N=26)	CMF (N=29)	UFT (N=29)
Patients, n (%)								
N•SAS-BC 01	157 (74.8)	150 (71.4)	52 (88.1)	62 (84.9)	35 (83.3)	20 (76.9)	22 (75.9)	23 (79.3)
CUBC	53 (25.2)	60 (28.6)	7 (11.9)	11 (15.1)	7 (16.7)	6 (23.1)	7 (24.1)	6 (20.7)
Age (years)								
Median (range)	52.0 (32–74)	52.0 (33–70)	53.0 (32–73)	52.0 (32–75)	52.5 (34–71)	55.0 (39–73)	54.0 (41–68)	56.0 (44–71)
<50, n (%)	90 (42.9)	90 (42.9)	21 (35.6)	25 (34.2)	15 (35.7)	12 (46.2)	5 (17.2)	5 (17.2)
≥50	120 (57.1)	120 (57.1)	38 (64.4)	48 (65.8)	27 (64.3)	14 (53.8)	24 (82.8)	24 (82.8)
Tumor size (cm)								
Median (range)	2.0 (0.7–8.0)	2.2 (0.5–9.5)	2.2 (0.9–8.0)	2.3 (0.5–7.8)	2.0 (0.5–4.5)	2.0 (0.5–10.0)	2.5 (0.7–6.0)	2.2 (0.9–5.0)
<3 cm, n (%)	161 (76.7)	150 (71.4)	40 (67.8)	51 (69.9)	35 (83.3)	17 (65.4)	19 (65.5)	20 (69.0)
≥3 cm	49 (23.3)	60 (28.6)	19 (32.2)	22 (30.1)	7 (16.7)	9 (34.6)	10 (34.5)	9 (31.0)
Lymph nodes, n (%)								
0	157 (74.8)	150 (71.4)	52 (88.1)	62 (84.9)	35 (83.3)	20 (76.9)	22 (75.9)	23 (79.3)
1–3	45 (21.4)	49 (23.3)	7 (11.9)	10 (13.7)	6 (14.3)	5 (19.2)	3 (10.3)	5 (17.2)
4	8 (3.8)	11 (5.2)	0	1 (1.4)	1 (2.4)	1 (3.8)	4 (13.8)	1 (3.4)
Histological type, n (%)								
Invasive ductal	188 (89.5)	186 (88.6)	55 (93.2)	65 (89.0)	42 (100.0)	25 (96.2)	29 (100.0)	26 (89.7)
Other	22 (10.5)	24 (11.4)	4 (6.8)	8 (11.0)	0	1 (3.8)	0	3 (10.3)
Estrogen receptor, n (%)								
Negative	5 (2.4)	4 (1.9)	59 (100.0)	73 (100.0)	1 (2.4)	0	29 (100.0)	29 (100.0)
Positive	205 (97.6)	206 (98.1)	0	0	41 (97.6)	26 (100.0)	0	0
Progesterone receptor, n (%)								
Negative	34 (16.2)	28 (13.3)	59 (100.0)	73 (100.0)	16 (38.1)	9 (34.6)	29 (100.0)	29 (100.0)
Positive	176 (83.8)	182 (86.7)	0	0	26 (61.9)	17 (65.4)	0	0
Ki67 (%)								
Median (range)	19.0 (0.0–90.8)	15.9 (0.0–83.7)	41.5 (0.0–97.2)	44.4 (0.0–86.0)	27.0 (0.0–69.7)	30.4 (2.4–57.3)	29.8 (0.0–60.7)	33.5 (0.0–67.7)
<20%	115 (54.8)	130 (61.9)	13 (22.0)	14 (19.2)	10 (23.8)	6 (23.1)	7 (24.1)	4 (13.8)
≥20%	95 (45.2)	80 (38.1)	46 (78.0)	59 (80.8)	32 (76.2)	20 (76.9)	22 (75.9)	25 (86.2)
Nuclear grade, n (%)								
Grade 1	81 (38.6)	85 (40.5)	4 (6.8)	2 (2.7)	2 (4.8)	3 (11.5)	2 (6.9)	0

Grade 2	59 (28.1)	76 (36.2)	7 (11.9)	5 (6.8)	10 (23.8)	6 (23.1)	4 (13.8)	2 (6.9)
Grade 3	70 (33.3)	49 (23.3)	48 (81.4)	66 (90.4)	29 (69.0)	17 (65.4)	23 (79.3)	27 (93.1)
Unknown	0	0	0	0	1 (2.4)	0	0	0
Histological grade, n (%)								
Grade 1	36 (17.1)	28 (13.3)	1(1.7)	0	1 (2.4)	2 (7.7)	0	0
Grade 2	109 (51.9)	136 (64.8)	14 (23.7)	9 (12.3)	15 (35.7)	7 (26.9)	8 (27.6)	4 (13.8)
Grade 3	65 (31.0)	46 (21.9)	44 (74.6)	64 (87.7)	25 (59.5)	17 (65.4)	21 (72.4)	25 (86.2)
Unknown	0	0	0	0	1 (2.4)	0	0	0
Tumor-infiltrating lymphocytes, n (%)								
Low	163 (77.6)	183 (87.1)	28 (47.5)	29 (39.7)	26 (61.9)	18 (69.2)	10 (34.5)	11 (37.9)
Intermediate	31 (14.8)	21 (10.0)	22 (37.3)	30 (41.1)	9 (21.4)	5 (19.2)	12 (41.4)	10 (34.5)
High	15 (7.1)	5 (2.4)	9 (15.3)	14 (19.2)	7 (16.7)	3 (11.5)	7 (24.1)	8 (27.6)
Unknown	1 (0.5)	1 (0.5)	0	0	0	0	0	0

CMF, cyclophosphamide, methotrexate, and fluorouracil; UFT, tegafur-uracil; HER2, human epidermal growth factor receptor 2; HR, hormone receptor

Supplementary Fig. 1 Kaplan–Meier survival curves for relapse-free survival (RFS) (a) and overall survival (OS) (b) adjusted for age, tumor size, nodal status, histological type, estrogen receptor (ER), and progesterone receptor (PgR) in all patients, and for RFS (c) and OS (d) adjusted for tumor size and nodal status in patients included in the final analysis. ER and PgR data used for the adjustment in all patients were measured at each study site, and those included in the final analysis were measured by central pathological review

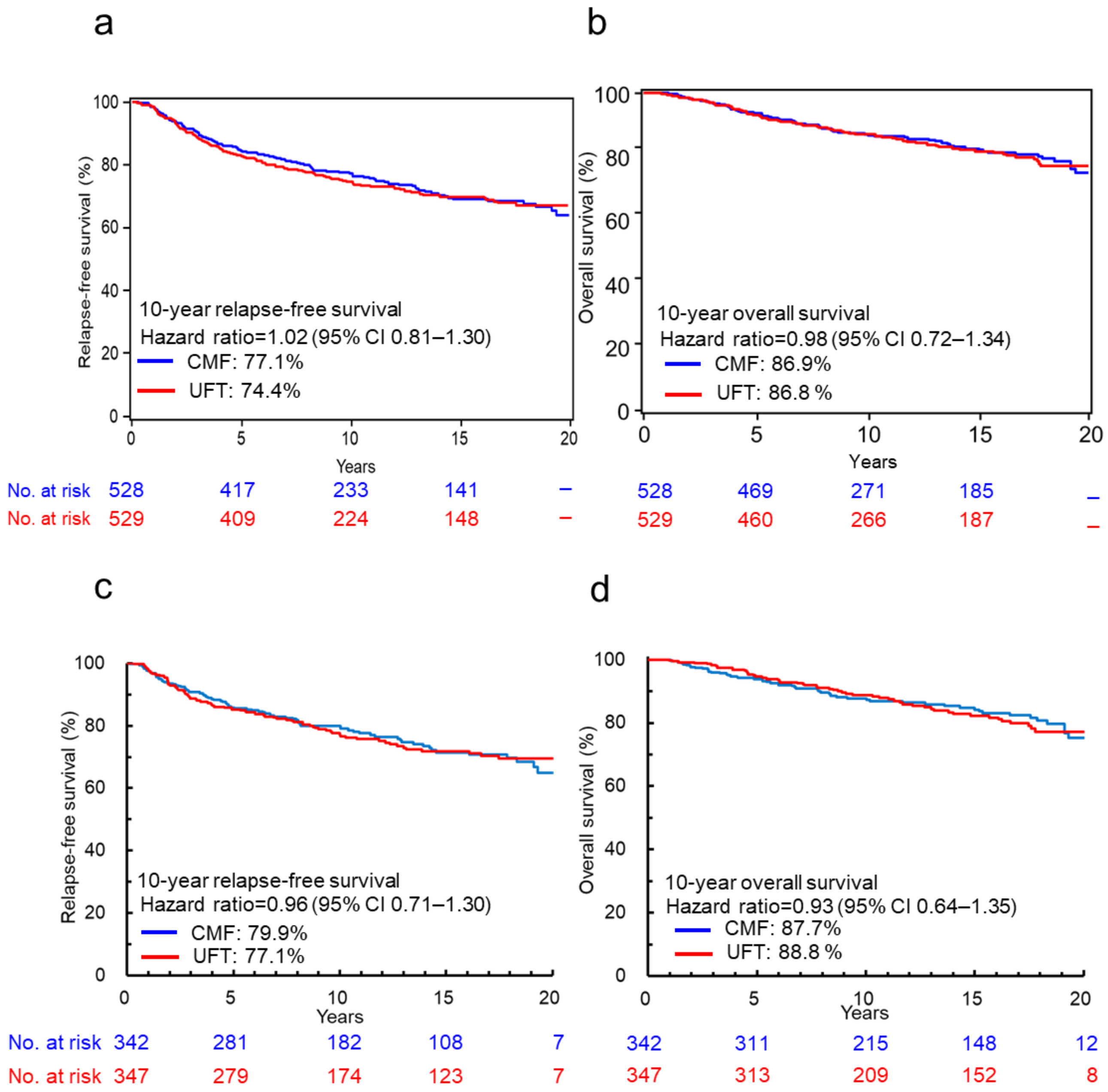

CI, confidence interval, CMF, cyclophosphamide, methotrexate, and fluorouracil; UFT, tegafur-uracil
